# Herb-Herb Combination for Therapeutic Enhancement and Advancement: Theory, Practice and Future Perspectives

**DOI:** 10.3390/molecules18055125

**Published:** 2013-05-03

**Authors:** Chun-Tao Che, Zhi Jun Wang, Moses Sing Sum Chow, Christopher Wai Kei Lam

**Affiliations:** 1 Department of Medicinal Chemistry and Pharmacognosy and WHO Collaborating Center for Traditional Medicine, College of Pharmacy, University of Illinois at Chicago, Chicago, IL 60612, USA; 2 Center for Advancement of Drug Research and Evaluation, College of Pharmacy, Western University of Health Sciences, Pomona, CA 91766, USA; 3 State Key Laboratory of Quality Research in Chinese Medicine, Macau Institute for Applied Research in Medicine and Health, Macau University of Science and Technology, Macau, China

**Keywords:** herb-herb combination, herb-herb interaction, Chinese medicine, multi-item prescription, synergy

## Abstract

Herb-herb combinations have been used in Chinese medicine practice for thousands of years, yet scientific evidence of their therapeutic benefits is lacking. With increasing interest in shifting from the one-drug-one-target paradigm to combination therapy or polypharmacy to achieve therapeutic benefits for a number of diseases, there is momentum to explore new knowledge by tapping the past empirical experiences of herb-herb combinations. This review presents an overview of the traditional concept and practice of herb-herb combination in Chinese medicine, and highlights the available scientific and clinical evidence to support the combined use of herbs. It is hoped that such information would provide a lead for developing new approaches for future therapeutic advancement and pharmaceutical product development. Very likely modern technologies combined with innovative research for the quality control of herbal products, identification of active components and understanding of the molecular mechanism, followed by well-designed animal and clinical studies would pave the way in advancing the wealth of empirical knowledge from herb-herb combination to new therapeutic modalities.

## 1. Introduction

Herbal products are of interest to many patients and health care practitioners since about 70% of population worldwide rely on herbal medicines for part of their primary health care [1]. In different regions and cultures, herbal products are used as single herb, combination of herbs, or combination of herb(s) and drug(s). When herbs are used in combination, the effects can be complicated as various interactions can occur among the individual components. The most desirable interactions are those which can result in additional therapeutic benefit. This is often the intended or expected outcome when using combination therapy. However, due to the presence of multiple components in the herbal products, the effects arising from herb-herb or herb-drug interactions are often unpredictable and complicated. Various types of pharmacokinetic and pharmacogenomic interactions from herb-drug combinations have been well described and documented in recent literature [2,3,4,5,6,7,8,9,10]. On the other hand, much less information is available on herb-herb interaction, although herb-herb combination has been used and documented as a desirable therapeutic approach in China since the time of the *Yellow Emperor’s Canon of Internal Medicine* (*Huangdi* *Neijing*) more than 2,000 years ago [11].

Currently, combination therapies are employed for the treatment of critical diseases, such as cancer, acquired immunodeficiency syndrome (AIDS) and pulmonary tuberculosis, in order to achieve enhanced therapeutic effects. The modern approach of combination therapy is a renewal of what was advocated in Chinese medicine that started thousands of years ago on the use of herb-herb combination for improvement of therapeutic outcome. Can the past experience of Chinese medicine provide new clue to identifying potential effective combinations for certain conditions that cannot be adequately treated by modern medicine? How can we best tap the wealth of past empirical experience to enhance future therapeutic advancement? Since very little has been written on the subject of herb-herb combination/interaction, we surveyed both English and Chinese literature from databases such as MEDLINE, EMBASE, the Traditional Chinese Medical Database system [12], and the Wanfang database [13] on this subject and compiled an overview of its theoretical principle and empirical use to provide an assessment of its role and future direction for therapeutic enhancement and advancement.

## 2. Theoretical Perspectives on Herb-Herb Combination

The concept and practice of Chinese medicine is derived from the accumulation of empirical evidence and subsequent deduction to form a series of theories, many of which were borrowed from philosophical thoughts such as the balance between *yin* and *yang* and the interrelationship among the “five elements (*wu-xing*)” within the body [11,14,15,16]. Thus the tenet of Chinese medicine is based on the theory that the occurrence and development of diseases are caused by an imbalanced status (e.g., excessive “coldness” or “heat”) in various body parts (e.g., the *zang*- and *fu*-organs), and the use of medicaments or other means such as acupuncture and physical manipulation can restore such imbalance so as to re-establish a state of equilibrium (homeostasis).

Accompanying the above theories of disease, a system of Chinese herbology was also developed to guide the selection of medicaments for the treatment of diseases [11,14,15,16,17]. The system provided the basis for prescription by Chinese medicine doctors. Most Chinese medicine prescriptions contain more than one medicinal plant (and/or animal/mineral substance) to form a multi-item concoction (*fu-fang*). When medications are prescribed, the principles of interactions among the herbs must be taken into consideration by the clinicians so as to compose an optimal prescription for therapeutic benefits. The concept of herb-herb interaction, based on the notions of positive (complementation) or negative (antagonism) outcomes, was therefore developed. Early in the history of use of medicinal agents, it was realized that the presence of one herb may alter the effect of the other when they are co-administered. The combined effect, either complementary or antagonistic, would be manifested in the clinical outcome. Take for example the Decoction of Ephedra (*Mahuang Tang*), which contains ephedra, cinnamon twig, bitter apricot seed, and licorice root. The prescription is used not only for its diaphoretic effect, but also for the relief of coughing and asthma, as well as reducing headaches and general aching during common cold [18]. All of these symptoms are interpreted by the Chinese medicine theory to be caused by excessive “coldness” and “wind” in the body. The co-administration of multiple ingredients would result in complementary interactions to combat symptoms of common cold or influence the illness. The scenario is comparable to the modern combination medicines with antipyretic, cough suppressant and nasal decongestant properties used in the treatment of common cold.

The Pulse-Activating Powder (*Shengmai San*) is another example of herbal combination. The formula consists of ginseng root, *Ophiopogon* (dwarf lily turf) root, and *Schisandra* (magnolia vine) fruit. Altogether, they are indicated for the deficiency syndrome of *qi*, *yin*, and body fluid, as manifested by profuse sweating, dryness of the mouth, shortness of breath, thirst, and faint pulse [19]. This three-item concoction is therefore used to invigorate the *qi*, nourish the *yin*, and promote the production of body fluid. It is “pulse-activating” because it can be used for emergency treatment of severe cases of *qi* and *yin* deficiency when the pulses become very weak. More often, the concoction is prescribed to restore *qi* and *yin* in patients suffering from chronic diseases. In this formula, ginseng root serves to strengthen *qi*, dwarf lily turf replenishes *yin*, whereas magnolia fruit takes up the roles of consolidating *qi* and *yin*, and arresting sweating. The traditional usage of the *Shengmai* formula has led to intensive studies of its cardiovascular pharmacology; and evidence is now emerging to indicate potential clinical applications in the treatment of cardiac diseases such as intradialytic hypotension [20], heart failure [21,22], and viral myocarditis [23].

Based on Chinese herbal medicine theory and practice, multi-item prescriptions are formulated deliberately according to six basic modes of herb-herb interactions; namely, reinforcement, potentiation, restraint, detoxification, counteraction, and toxicity. They serve as guiding principles when multi-item concoctions are prescribed in order to enhance the safe and effective use of the herbs.

### 2.1. Reinforcement

Reinforcement refers to situations in which herbs possessing similar medicinal properties are used together to produce a greater efficacy. Taking the Decoction of Ephedra as an example, ephedra and cinnamon twig form a pair of diaphoretic agents; the adrenergic (sweating) effect of the former is strengthened by the latter through an increase of peripheral blood flow. As a result, the febrifugal effect is enhanced. Another example of mutual reinforcement is the combination of the rhizomes of *Corydalis yanhusuo* (*yan-hu-suo*) and *Curcuma phaeocaulis* (*er-zhu*) in the ancient formula *Yanhusuo San*, which is useful for improving blood circulation, alleviating blood stasis, and relieving pain. The action of *Corydalis* rhizome is to promote *qi* movement in the body so as to reinforce the blood circulation effect of *Curcuma phaeocaulis*. Another reinforcement pair, honeysuckle (*Lonicera japonica*) flower and *Forsythia* *suspense* fruit, is often prescribed together to eliminate heat and wind, as well as the toxic components from the body, for the treatment of common cold or influenza. From a modern pharmacological point of view, reinforcement can probably be interpreted to include either an additive effect (when the combined effect of two herbs equals the sum of the effect of each agent given alone) or synergistic effect (when the combined effect exceeds the sum of the effects of individual herbs). A similar concept in modern medicine is the use of “cocktail” in antiretroviral therapy (HAART) [24]. The usual HAART regimen combines three or more different drugs such as reverse transcriptase inhibitors and protease inhibitors in order to optimize antiviral efficacy.

### 2.2. Potentiation

Potentiation refers to situations when two herbs are used in the same prescription, in which one of them serves as the principal herb and the other an adjunct or auxiliary herb. The role of the auxiliary herb is to help strengthen the effect of the principal herb. An example is the combination of ginseng root and aconite (*Aconitum carmichaelii*) daughter root in an anti-shock remedy, the Decoction of Ginseng and Aconite (*Shenfu Tang*). In the formula, ginseng is the principal component to strengthen the *primary-qi* (energy and vitality) in order to resuscitate, whereas aconite helps to “warm” the body and raise the *yang* energy. Working together, they are able to replenish both inborn and postnatal (acquired) essence (vitality) in the body and to restore vital physiological functions particularly during the emergency condition of collapse and shock [25]. Potentiation of the therapeutic efficacy is also demonstrated in the Shaoyao Gancao Decoction, which is composed of the roots of paeony (*Paeonia* *lactiflora*) and licorice (*Glycyrrhiza* *uralensis*) for the treatment of pain (e.g., dysmenorrhea, muscular spasm, and irritable bowel). A recent study in a rat neuropathic pain model has demonstrated the synergistic effect of a combination of paeony glycosides and licorice flavonoids in the ratio of 3:1 [26]. In modern medicine, a similar concept of potentiation has been developed to make use of pharmaco-enhancing agents (or so-called “booster” drugs) in anti-HIV regimens [27], in which the boosterdrug interacts with the antiviral drug pharmacokinetically to slow down the liver metabolism of the latter by liver enzymes, thereby raising the active drug concentration in blood to improve and prolong the therapeutic effect.

### 2.3. Restraint and Detoxification

In Chinese medicine, many herbs are considered to possess toxic properties. The concept of toxicity refers to different degrees of adverse or undesirable effects, but not necessarily fatality. Therefore the use of so-called “toxic herbs,” *i.e.*, herbs with toxic property, for therapeutic purposes is acceptable or even desirable in some instances. As a matter of fact, the use of toxic herbs is preferred in the treatment of certain severe and critical conditions such as tumor (anti-tumor agents), severe infection (anti-bacterial and anthelmintic agents), and cardiovascular abnormality (cardiotonic and anti-arrhythmic agents) [28]. For example, *Tripterygium wilfordii* (*lei-gong-teng*), a toxic plant traditionally used for “dispelling wind”, “removing dampness”, and reliving pain, has now shown promising clinical potential for the treatment of rheumatoid arthritis and other immune diseases [29]. Two of its chemical ingredients, triptolide and celastrol, have also found potential applications in the treatment of inflammatory disorders and cancers [30,31,32,33]. When toxic herbs are used, combination with other herbs is sometimes taken as a means to make the toxic effect more tolerable. A toxic herb is said to be “restrained” by a non-toxic partner, which is capable of “detoxifying” the former to alleviate the toxic symptoms. In another words, the non-toxic partner acts like an antidote to reduce or neutralize the toxicity. A typical example of the “restraint” and “detoxification” effect is the combined use of ginger and pinellia (*Pinellia* *ternata*) tuber. Pinellia tuber is considered a toxic herb that would cause severe mucosal irritation and inflammation in the throat and the gastrointestinal tract. Calcium oxalate and agglutinin have been suggested to be the toxic principles causing inflammatory responses [34,35,36,37]. In order to reduce the toxicity of pinellia (without compromising its therapeutic effect), it is often cooked together with ginger. In fact, an animal study has shown that ginger can reduce the incidence of abdominal torsion in mice after intra-peritoneal injection of pinellia; it also suppressed the capillary permeability in the abdominal cavity as well as reduced prostaglandin E_2_ contents in the inflammatory foot tissue of mice after injection of pinellia [38]. While the exact mechanism of the detoxifying action between the herbs is unknown, it can be speculated that there may be alteration in the solubility of chemical ingredients, slowing down the absorption of the toxic ingredients, and/or structural changes of the toxic ingredients. The main utility of this mode of interaction is therefore to make use of the partner herb to restrain the toxic effect, but not affecting the main therapeutic action of the toxic herb.

### 2.4. Counteraction

Counteraction refers to situations in which the therapeutic effect of an herb is diminished by another herb. This can be interpreted as an antagonistic interaction, although in traditional Chinese medicine, the concept of physical, chemical, dispositional, or receptor antagonism is lacking. A well-known example of counteraction is the interaction between turnip root/seed and ginseng root. Ginseng is often used as a tonic drug to elevate the vital energy (*qi*), but when used in the presence of turnip, its *qi*-tonifying effect would be greatly reduced or even eliminated. Interestingly, such counteraction has been observed in a recent study which found turnip juice capable of abolishing the genoprotective effect of ginseng [39]. Although the exact mechanism remains unknown, the knowledge of herb-herb counteraction can serve as a guide for Chinese herbal pharmacy practice when herbal combinations are prescribed.

### 2.5. Incompatibility

Another herb-herb interaction leading to unfavorable outcome is the situation where combination would result in toxic or severe adverse effects, *i.e.*, the herbs are mutually incompatible and therefore should be avoided in all prescriptions. In the Chinese medicinal literature, there are unambiguous stipulations of the so-called “Eighteen antagonistic medicaments” and the “Nineteen contraindicating medicaments” (Table 1). While the exact pharmacological mechanisms of such interactions are not clearly understood, there are *in vitro* studies on the effect of incompatible pairs such as *Glycyrrhiza*/*Daphne genkwa*, *Glycyrrhiza*/*Euphorbia kansui*, *Sophora* *flavescens*/*Veratrum* *nigrum* and pinellia/aconite relating to the induction or inhibition of cytochrome P-450 enzymes (CYP1A2, CYP3A1/2 and CYP2E1) [40,41,42,43]. Along the same line, ginseng root has been observed to suppress the enzyme activity and mRNA expression of CYP450 isozymesin the presence of *Veratrum* *nigrum* root [44]. Further studies may offer insight into the possible mode of clinical interaction in the future.

**Table 1:** Chinese traditional knowledge of incompatible herbal drug pairs:the “eighteen antagonistic medicaments” and the “nineteen contraindicating medicaments”.

**The Eighteen Antagonistic Medicaments**	
***Glycyrrhiza* root (licorice) is antagonistic to:**	*Euphorbia pekinensis* root
	*Euphorbia kansui* root
	*Daphne genkwa* flower
	*Sargassum* spp. (seaweed)
***Aconitum carmichaelii* root (aconite) is antagonistic to:**	*Fritillaria* spp. bulb
	*Trichosanthes* *kirilowii* fruit
	*Pinellia ternate* rhizome
	*Ampelopsis japonica* root
	*Bletilla striata* rhizome
***Veratrum*** ***nigrum* root is antagonistic to:**	*Panax ginseng* (ginseng) root
	*Salvia miltiorrhiza* root
	*Adenophora* *stricta* root
	*Scrophularia* *ningpoensis* root *
	*Sophora* *flavescens* root
	*Asarum sieboldii* herb
	*Paeonia lactiflora* root
**The Nineteen Contraindicating Medicaments**	
**Suphur is contraindicated to:**	Sodium sulphate
**Mercury is contraindicated to:**	Arsenic trioxide
***Euphorbia ebracteolata* root is contraindicated to:**	Litharge
***Croton tiglium* seed is contraindicated to:**	*Pharbitis* *purpurea* seed
***Eugenia caryophyllata* flower bud is contraindicated to:**	*Curcuma* spp. root
**Sodium sulphate is contraindicated to:**	*Sparganium* *stoloniferum* rhizome
***Aconitum carmichaelii* and *A. kusnezoffii* roots are contraindicated to:**	*Rhinoceros* horn
***Panax ginseng* root is contraindicated to:**	*Trogopterus* faeces
***Cinnamomum cassia* bark is contraindicated to:**	Red halloysite

* This herb is an additional item included in the Eighteen Antagonistic Medicament in some literature.

## 3. Herb-Herb Interaction in Chinese Medicine Practice

In Chinese medicine practice, a special concept of herb-herb interaction is utilized when multi-item prescriptions are formulated. Although each individual herb included in a prescription is indicated for certain symptom(s), when multiple herbs are administered together, the mixture is more than a random group of herbs. Rather, they work in unison through mutual interaction resembling different roles of the government official hierarchy, with each herb figuratively designated as either “emperor”, “minister”, “assistant”, or “servant” [14]. The following provides a description and examples of the four hierarchical roles of herbs in prescription practice.

*The “Emperor” Herb*: In ancient Chinese government structure, the emperor is the most important and authoritative figure at the top of hierarchy. An analogy for the emperor herb in a multi-item prescription is its designated role to handle the main course of disease or deal with major symptoms. An emperor herb is sometimes translated as the King or Monarch. Take the Decoction of Ephedra as an example for treating common cold (particularly the “exterior-cold type” as defined by the traditional theories), ephedra plays the leading role as an emperor to induce perspiration, relieve coughs, and smooth the flow of *qi*. Its pharmacological effects can be attributed to ephedrine and related alkaloids, which possess adrenalin-like and sympathomimetic properties. 

*The “Minister” Herb*: The minister herb serves two roles -it is either an adjunct to the emperor herb to reinforce and support, and/or it is used to deal with certain minor symptoms of the disease. In the Decoction of Ephedra, the cinnamon twig takes up the role of a minister. This herb is thought to have the ability to “warm the channel”so as to dispel excessive “coldness” from the body, and expel “evils” (the pathogenic factors) from the muscles and skin so as to relieve common cold symptoms. Pharmacological studies have revealed that cinnamon twigs can increase sweating by dilating peripheral blood vessels and ease pains in the limbs during common cold.

*The “Assistant” Herb*: Assistant herb, also known as the Clerical, Adjuvant, or Auxiliary herb, may play three roles. First, it can help to optimize the therapeutic effects of the emperor and minister. Second, it can be used to diminish or counteract the toxicity caused by the emperor or minister, thereby reducing the undesirable effects of the entire prescription and tempering the drastic action of the principal herbs. Third, it can serve to maintain a balance such as the warm-cold effect. While the assistant herb plays a relatively minor role itself, it is an essential component in a multi-herb concoction. To cite the example of the Decoction of Ephedra, apricot seed acts as the assistant, helping to ventilate the lungs and stopping cough during common cold. 

*The “Servant” Herb*: The servant herb, sometimes referred to as the Convoy, Dispatcher, Guide, or Emissary drug, is perceived to play the role of a guide or courier so as to direct the essence of the entire prescription to the target organ/tissue or meridian. It may also serve as a harmonizer or moderator to “bind” the formula together. Licorice root plays the role of a servant in the Decoction of Ephedra to regulate the overall medicinal property of the prescription. 

From a modern pharmacological point of view, interactions among herbs in a multi-item prescription can occur either pharmacokinetically or pharmacodynamically. Furthermore, Chinese medicine often provides multi-target (polyvalent) treatment with individual components affecting different symptoms and at different target organs. Such a multi-target interaction is consistent with the modern concept of polypharmacy.

In summary, the concept and practice of herb-herb combination has been recognized in Chinese medicine for thousands of years. The rationale of the multi-item prescription appears to be contrived when judging from scientific perspective, but their empirical effects are obvious. While the exact pharmacological mechanisms are not clearly understood, pharmacokinetic, pharmacodynamic, and/or polyvalent effects are likely involved.

## 4. Scientific and Clinical Studies on Herb-Herb Combinations

Despite the wealth of experience with multi-herb prescription in Chinese medicine practice, scientific efforts to understand herb-herb interaction or proof of clinical benefit of many such combinations have been slow and weak. One reason may be due to the immense undertaking and high cost involved in conducting clinical trials on herbal products. The most important reason, perhaps, is because the medical and pharmaceutical communities have adopted the concept and philosophy of the “silver bullet” approach of therapy [45], and drug discovery and development programs carried out by the pharmaceutical industry have been very much geared to the “single ingredient” and “one-drug-one-target” paradigm.

With increasing acceptance of combination therapy for the treatment of diseases such as cancer, AIDS and others [46,47,48], scientific interest to study the pharmacological effects of multiple-componentherbal preparations or multi-item prescriptions is expected. The interest in studying herbal products is further supported by the observations that many herbal extracts show superior effect when compared to single chemical constituents at the equivalent dose (or concentration). Williamson was one of the first to address such observations in a review article, in which examples of herbal interactions using *in vitro* and animal models as well as clinical observations were highlighted [49]. Such interactions were referred to as “synergy”. Later, Houghton explained the pharmacological activity of complex herbal products by distinguishing the roles of synergy and polyvalence [50]. According to Houghton, synergy is referred to a situation in which only one pharmacological function is involved, whereas polyvalence is referred to a range of biological activities resulting in an overall enhancement of the pharmacological or clinical outcome. Wagner and Ulrich-Merzenich elaborated on the concept of synergy by describing examples of how molecular-biological methods could enable better understanding of the synergistic mechanisms underlying the interactive effects [51]. Four mechanisms were suggested: (1) Synergistic multi-target effects on enzymes, substrates, metabolites, receptors, ion channels, transport proteins, DNA/RNA, ribosomes, antibodies, *etc.*; (2) Pharmacokinetic or physiochemical effects based on improved solubility, resorption rate, and enhanced bioavailability; (3) Antagonistic interactions with resistance mechanisms of pathogenic microorganisms; and (4) Elimination or neutralization of adverse effects caused by other substances present in the mixture.

Further examples of scientific studies demonstrating synergistic effects include:

(1) More potent antispastical effect was observed from cannabis extract (containing equimolar tetrahydrocannabinol content) than from tetrahydrocannabinol alone, suggesting involvement of other chemical ingredients present in the plant extract [52];
(2) Synergistic interaction between total glycosides of *Paeonia* root and total flavonoids of *Glycyrrhiza* root on neuropathic pain was demonstrated in rats using isobolographic analysis [26];
(3) Significantly higher anti-proliferative effect in HT-29 and CCD-18Co cell lines was found from *Angelica sinensis* extract than the corresponding mixture of three main phthalide constituents, *n*-butylidenephthalide, senkyunolide A, and *z*-ligustilide, suggesting some other ingredients were involved in enhancing the efficacy of the crude extract [53];
(4) More potent antidepressant activity from a combination of the active ingredients of *Rhodiola* *rosea* (*i.e.*, rhodioloside, rosavin, rosarin, and rosin) was observed than from each individual component when evaluated in a forced swimming rat model [54];
(5) A combination of the rhizomes of *Corydalis yanhuosuo* and *Curcuma phaeocaulis* at a ratio of 3:2 exhibited the strongest anti-proliferative effect on the MDA-MB-231 human breast cancer cell line [55];
(6) A combination of the roots of *Astragalus* *membranaceus* and *Rehmannia* *glutinosa* (in 2:1 ratio) significantly reduced the wound area of rats in a foot ulcer animal model, whereas no wound-healing effect was observed when individual herb was applied to the wound [56];
(7) The oral bioavailability of geniposide in rats was dramatically enhanced in the presence of *Citrus aurantium* and *Magnolia officinalis*, suggesting that herb-herb interaction may have led to a better bioavailability of the active ingredient [57];
(8) When administered in the form of *Artemisia* tea, the active ingredient artemisinin was found to be more rapidly absorbed than when it was given in the form of pure compound [58].

Besides the above pharmacological studies, at least two clinical studies demonstrating the therapeutic benefits of herb-herb combination are available. Wheatley reported positive results of an herbal combination in a small-scale, open, cross-over trial in patients suffering from stress-induced insomnia [59]. The patients first received six-week treatment with kava-kava (*Piper methysticum*); after an intervening wash-out period of two weeks, the trial continued with valerian (*Valerian aofficinalis*) for six weeks. After another two-week washout, the patients further received a combination of kava-kava and valerian for six weeks. The trial showed that the combination treatment during the last six weeks significantly improved sleep. In another example, Scholey and Kennedy conducted a double-blind, placebo-controlled study on the performance of healthy adults following single dose of *Ginkgo biloba* or ginseng, as well as their combination using a computerized test of serial subtraction tasks (“Serial Three” and “Serial Seven”) [60]. Specifically, an instruction screen informed the participant to count backwards in threes or sevens from a given number, as quickly and accurately as possible by using the numeric keypad to enter response. A highly significant and sustained improvement of responses following treatment with the Ginkgo-ginseng combination was observed.

In summary, in the last decade or so, an encouraging number of scientific and clinical studies have begun to provide preliminary understanding and evidence of enhanced efficacy of herb-herb combination.

## 5. Challenges

The wealth of knowledge from empirical use of herb-herb combination according to Chinese medicine concepts described above has not been systematically explored to benefit modern pharmacotherapy. Since only limited scientific and clinical studies have been carried out so far, a great potential exists to probe the potential therapeutic benefit from the herb-herb combinations used in Chinese medicine practice. For reaping such benefit, we believe that improving future herbal research in the following areas is essential.

### 5.1. Improved Quality Control Methods

One of the fundamental requirements for medicinal products is quality assurance/control (QA/QC), without which scientific and clinical studies would be greatly hampered and less likely to yield valid results. At present the QA/QC of herbal products are inadequate. The current approaches to standardize herbal preparations include botanical verification, chromatographic profiling of chemical components, as well as quantification of selected marker components (which, in many cases, could be pharmacologically inert or biologically inactive) [61]. DNA fingerprinting and bar-coding have been applied for authentication purpose [62,63], but the molecular approach does not necessarily reflect or guarantee the pharmacological or clinical potency of the herbs. The situation becomes much more complicated and challenging for multi-herb products, in which hundreds, if not thousands, of chemical components may be present. Not only that our knowledge of the structural composition of these components is lacking, but more importantly, the information on the pharmacological activities of each chemical component is incomplete. Therefore, a “break-through” from the conventional approaches will be needed for proper QA/QC of herbal products. In this respect, development of more powerful approaches capable of defining the integrity of complex mixtures, such as the “omics” and systems biology technologies, should be investigated. Technologies such as expression patterns at the genomic, proteomic, and metabolomic levels are already available and could open new revenue to define both the composition and biological responses of complex multi-component mixtures and allow us to probe into the chemical-biological standardization of herbal products. 

A recent proposed method of quality control for botanical formulation is the technique of PhytomicsQC, which integrates the use of liquid chromatography/mass spectrometry (LC/MS) for chemical characterization and chemical fingerprinting, differential cellular gene expression for bioresponse fingerprinting, and animal pharmacology for *in vivo* validation [64]. Procedures to determine and compare the similarity of the chemical and bioresponse fingerprints among different manufactured batches were proposed. On the other hand, a biological quality control assessment method called Biological Response Fingerprinting (BioReF) is a genome-wide approach to make use of a set of marker genes that define a signature pattern for a specific botanical formulation [65]. The expression of these marker genes represents the biological responses of human cells to the chemical components of the botanical drug; and the pattern was suggested to serve as a means for QC assessment in terms of the consistency of biological activities. In addition, pharmacokinetic and geonomic approach to identify and standardize an herbal mixture product, the Shiwu Tang, has also been demonstrated [66]. Such technologies together with functional fingerprints may lead to an improved approach for future QA/QC of herbal products.

### 5.2. Understanding Herb-Herb Interactions at the Molecular, Cellular, and Organism Levels

In Chinese medicine practice, while many multi-item prescriptions have been used clinically for thousands of years, their exact pharmacological actions remain unclear and should be determined in order to validate and better understand the empirical observations. However, the routine pharmacological protocols are likely to be inadequate to demonstrate the unique composite effect of herb-herb combination. The use of systems biology and the “omic” technologies may provide a new approach to evaluate the synergistic effects of phytopharmaceuticals [67,68,69,70,71]. To emphasize the important roles of systems biology in herbal science, Sarris et al. have coined a term “herbomics” to describe the “omic” studies of herbs [72]. This approach is particularly attractive for understanding the profiling of multiple gene and protein targets.

Combination of molecular, cellular and analytical techniques may be utilized to explore and better describe the effects of multi-item prescriptions. For example, the design of a Chinese medicinal formula containing realgar, *Indigo naturalis*, and *Salvia miltiorrhiza* for treating promyelocytic leukemia was rationalized by a systems biology approach [73]. In this study, three major chemical ingredients (one taken from each component of the formulation), namely, tetraarsenic tetrasulfide (from realgar), indirubin (from indigo), and tanshinone IIA (from *Salvia*), were combined and tested for *in vivo* and *in vitro* activities. The mixture was found to yield synergy in the treatment of leukemia in mice and in the induction of promyelocytic leukemia cell differentiation both *in vivo* and *in vitro*. There was also involvement of a number of cell cycle modulators, oncoproteins, and cell differentiation regulators in a concerted manner.

There is also a need to analyze large volumes of research data generated from the evaluation of multi-component herbs and synergistic effects [74,75,76]. An attempt to make use of the computer-aided approach such as the “network target-based identification of multicomponent synergy” (NIMS) has been proposed to identify synergistic drug combinations at the molecular level. It is hoped that an accurate analysis of such data can lead to new discovery of active componentsand help elucidate the mechanisms associated with many of the multi-item prescriptions empirically used for thousands of years.

### 5.3. Clinical Trials on Herbal Products

Once the components and mechanism of a given herb-herb-combination are identified, a quality product can then be prepared for testing the therapeutic effect of the product. The therapeutic effect must be carefully evaluated by a well-designed, randomized, double-blinded, placebo-controlled clinical trial involving a significant number of subjects (*i.e.*, with sufficient power). Unfortunately, such rigorous clinical studies are seldom performed due to high cost and other reasons. Small clinical studies are less costly but often yield negative or equivocal results, since the QC, mechanisms, and dose response are seldom considered in designing such studies. In the future, studies with questionable quality, small sample size, low level of evidence, and/or methodological weakness, all of which can lead to unreliable and inconclusive outcomes, should be avoided [77,78,79,80,81,82]. Instead, clinical trials following good clinical practice (GCP) should be implemented to demonstrate clinical efficacy of herb-herb combination. 

## 6. Conclusions

Despite vast experience from empirical use of herb-herb combination in Chinese medicine practice over two thousand years, well documented beneficial outcomes are lacking, and the six basic modes of Chinese medicine theory of interaction have not been adequately proven or confirmed by modern scientific studies. Nevertheless, the huge experience accumulated from clinical observations of herb-herb combination may provide a lead for future exploration of new therapeutic benefits and product development. Innovative research for better quality control of herbs, identification of active components, and understanding of the molecular mechanism, followed by well-designed animal and clinical studies will be needed to harness such benefits.
